# Supplementary material for: Colony defence in bumblebees (Bombus terrestris)
Source: PLoS One. 2025 Dec 3;20(12):e0335136. doi: 10.1371/journal.pone.0335136 (PMC12674513; doi:10.1371/journal.pone.0335136)
Supplement: S1 File — This document contains the tables S1 to S10 and their captions. (PDF) [file pone.0335136.s001.pdf]

# Colony defence in bumblebees (*Bombus terrestris*)

Sajedeh Sarlak[1], Divya Ramesh[2], Ahmad Ashouri[1], Seyed Hossein Goldansaz[1], Alica Schwarz[2], Lena Seitz[2], Anja Weidenmüller[2], Vlad Demartsev[2,3,4], Christoph Kleineidam[2,3], Morgane Nouvian[2,3,5*]

[1] Department of Plant Protection, College of Agriculture and Natural Resources, University of Tehran, Karaj, Iran

[2] Department of Biology, University of Konstanz, Konstanz, Germany

[3] Center for the Advanced Study of Collective Behaviour, University of Konstanz, Konstanz, Germany

[4] Department for the Ecology of Animal Societies, Max Planck Institute of Animal Behavior, Konstanz, Germany

[5] Zukunftskolleg, University of Konstanz, Konstanz, Germany

* E-mail: morgane.nouvian@uni-konstanz.de

## Supplementary information

**S1 Table.** Pairwise comparisons of bee velocity by time bin and bee type.

| Comparison | Estimate | SE | z-ratio | p-value |
|---|---|---|---|---|
| **Queens** | | | | |
| **Before – Acute Response** | –1.906 | 0.332 | –5.738 | <0.0001 *** |
| **Before – Delayed Response** | –0.085 | 0.334 | –0.255 | 0.9942 ns |
| **Before – Recovery** | 0.700 | 0.338 | 2.072 | 0.1624 ns |
| **Acute – Delayed Response** | 1.821 | 0.329 | 5.529 | <0.0001 *** |
| **Acute – Recovery** | 2.606 | 0.331 | 7.871 | <0.0001 *** |
| **Delayed Response – Recovery** | 0.785 | 0.330 | 2.383 | 0.0804 ns |

| | | | | |
|---|---|---|---|---|
| **Workers** | | | | |
| **Before – Acute Response** | −1.278 | 0.147 | −8.683 | <0.0001 *** |
| **Before – Delayed Response** | −0.427 | 0.147 | −2.902 | 0.0194 ** |
| **Before – Recovery** | 0.217 | 0.147 | 1.474 | 0.4531 ns |
| **Acute – Delayed Response** | 0.851 | 0.147 | 5.788 | <0.0001 *** |
| **Acute – Recovery** | 1.495 | 0.147 | 10.136 | <0.0001 *** |
| **Delayed Response – Recovery** | 0.644 | 0.147 | 4.370 | 0.0001 *** |
| **Between Bee Types** | | | | |
| **Before Disturbance (Queen – Worker)** | −1.369 | 0.260 | −5.269 | <0.0001 *** |
| **Acute Response (Queen – Worker)** | −0.741 | 0.255 | −2.906 | 0.0037 ** |
| **Delayed Response (Queen – Worker)** | −1.711 | 0.255 | −6.709 | <0.0001 *** |
| **Recovery (Queen – Worker)** | −1.852 | 0.256 | −7.225 | <0.0001 *** |

**S2 Table.** Pairwise comparisons of the probability of being outside the brood area across time bins.

| Comparison | Estimate | SE | z-ratio | p-value |
|---|---|---|---|---|
| **Before Disturbance – Acute Response** | −3.051 | 0.365 | −8.368 | <0.0001 *** |
| **Before Disturbance – Delayed Response** | −2.183 | 0.329 | −6.638 | <0.0001 *** |
| **Before Disturbance – Recovery** | −0.515 | 0.309 | −1.669 | 0.3403 ns |

| | | | | |
|---|---|---|---|---|
| **Acute Response – Delayed Response** | 0.868 | 0.295 | 2.939 | 0.0174 ** |
| **Acute Response – Recovery** | 2.536 | 0.340 | 7.464 | <0.0001 *** |
| **Delayed Response – Recovery** | 1.667 | 0.307 | 5.437 | <0.0001 *** |

**S3 Table.** Pairwise comparisons of the duration of being outside the brood area across time bins.

| **Comparison** | **Estimate** | **SE** | **z-ratio** | **p-value** |
|---|---|---|---|---|
| **Before Disturbance – Acute Response** | -0.4522 | 0.146 | -3.107 | 0.0102 ** |
| **Before Disturbance – Delayed Response** | 0.2857 | 0.147 | 1.950 | 0.2074 ns |
| **Before Disturbance – Recovery** | 0.3818 | 0.164 | 2.325 | 0.0923 ns |
| **Acute Response – Delayed Response** | 0.7379 | 0.104 | 7.071 | <0.0001 *** |
| **Acute Response – Recovery** | 0.8340 | 0.132 | 6.332 | <0.0001 *** |
| **Delayed Response – Recovery** | 0.0961 | 0.129 | 0.743 | 0.8797 ns |

**S4 Table.** Effects of disturbance on the probability of behaviours across time bins.

| **Behaviour** | **Comparison** | **Estimate** | **SE** | **z-ratio** | **p-value** |
|---|---|---|---|---|---|
| **Flying-Climbing** | **Before Disturbance – Acute Response** | -2.70 | 0.551 | -4.897 | <0.0001 *** |
| | **Before Disturbance – Delayed Response** | -1.71 | 0.543 | -3.148 | 0.0089 ** |

| | | | | | |
|---|---|---|---|---|---|
| | Before Disturbance – Recovery | ~0 | 0.636 | 0.000 | 1.0000 ns |
| | Acute Response – Delayed Response | 0.99 | 0.370 | 2.683 | 0.0367 * |
| | Acute Response – Recovery | 2.70 | 0.551 | 4.897 | <0.0001 *** |
| | Delayed Response – Recovery | 1.71 | 0.543 | 3.148 | 0.00 *** |
| **Patrolling** | Before Disturbance – Acute Response | -7.46 | 0.970 | -7.689 | <0.0001 *** |
| | Before Disturbance – Delayed Response | -4.68 | 0.947 | -4.947 | <0.0001 *** |
| | Before Disturbance – Recovery | -1.19 | 1.041 | -1.146 | 0.6609 ns |
| | Acute Response – Delayed Response | 2.77 | 0.316 | 8.777 | <0.0001 *** |
| | Acute Response – Recovery | 6.26 | 0.648 | 9.660 | <0.0001 *** |
| | Delayed Response – Recovery | 3.49 | 0.613 | 5.696 | <0.0001 *** |
| **Continuous Buzzing** | Before Disturbance – Acute Response | -45.8 | 15.23 | -3.004 | 0.0142 ** |
| | Before Disturbance – Delayed Response | -22.8 | 15.18 | -1.504 | 0.4351 ns |
| | Before Disturbance – Recovery | -13.7 | 15.33 | -0.895 | 0.8073 ns |
| | Acute Response – Delayed Response | 22.9 | 3.05 | 7.517 | <0.0001 *** |
| | Acute Response – Recovery | 32.0 | 4.15 | 7.719 | <0.0001 *** |

| | | | | | |
|---|---|---|---|---|---|
| | **Delayed Response – Recovery** | 9.1 | 1.78 | 5.107 | <0.0001 *** |
| **Leg Raising (Fisher's Test)** | **Before Disturbance – Acute Response** | - | - | - | <0.0001 *** |
| | **Before Disturbance – Delayed Response** | - | - | - | 1.0000 ns |
| | **Before Disturbance – Recovery** | - | - | - | 1.0000 ns |
| | **Acute Response – Delayed Response** | - | - | - | <0.0001 *** |
| | **Acute Response – Recovery** | - | - | - | <0.0001 *** |
| | **Delayed Response – Recovery** | - | - | - | 1.0000 *** |
| **Abdominal Pumping** | **Before Disturbance – Acute Response** | -295.1 | 6.15 | -47.953 | <0.0001 *** |
| | **Before Disturbance – Delayed Response** | -280.8 | 5.93 | -47.375 | <0.0001 *** |
| | **Before Disturbance – Recovery** | -252.2 | 6.11 | -41.293 | <0.0001 *** |
| | **Acute Response – Delayed Response** | 14.3 | 2.28 | 6.279 | <0.0001 *** |
| | **Acute Response – Recovery** | 42.9 | 3.70 | 11.587 | <0.0001 *** |
| | **Delayed Response – Recovery** | 28.6 | 2.93 | 9.776 | <0.0001 *** |
| **Perching** | **Before Disturbance – Acute Response** | -3.14 | 0.401 | -7.840 | <0.0001 *** |
| | **Before Disturbance – Delayed Response** | -1.96 | 0.357 | -5.491 | <0.0001 *** |

| | | | | | |
|---|---|---|---|---|---|
| | **Before Disturbance – Recovery** | 1.21 | 0.483 | 2.513 | 0.0579 |
| | **Acute Response – Delayed Response** | 1.18 | 0.290 | 4.075 | 0.0003 *** |
| | **Acute Response – Recovery** | 4.36 | 0.522 | 8.346 | <0.0001 *** |
| | **Delayed Response – Recovery** | 3.18 | 0.481 | 6.602 | <0.0001 *** |
| **Pulse Buzzing** | **Before Disturbance – Acute Response** | -0.856 | 0.326 | -2.628 | 0.0427 * |
| | **Before Disturbance – Delayed Response** | -2.380 | 0.339 | -7.030 | <0.0001 *** |
| | **Before Disturbance – Recovery** | -1.264 | 0.323 | -3.912 | 0.0005 *** |
| | **Acute Response – Delayed Response** | -1.524 | 0.295 | -5.174 | <0.0001 *** |
| | **Acute Response – Recovery** | -0.408 | 0.287 | -1.420 | 0.4869 ns |
| | **Delayed Response – Recovery** | 1.116 | 0.280 | 3.982 | 0.0004 *** |
| **Grooming** | **Before Disturbance – Acute Response** | 0.635 | 0.249 | 2.545 | 0.0532 * |
| | **Before Disturbance – Delayed Response** | -1.996 | 0.299 | -6.665 | <0.0001 *** |
| | **Before Disturbance – Recovery** | -0.625 | 0.247 | -2.531 | 0.0553 ns |
| | **Acute Response – Delayed Response** | -2.630 | 0.314 | -8.374 | <0.0001 *** |
| | **Acute Response – Recovery** | -1.260 | 0.258 | -4.883 | <0.0001 *** |

| | Delayed Response – Recovery | 1.371 | 0.294 | 4.659 | <0.0001 *** |
|---|---|---|---|---|---|

**S5 Table.** Effects of disturbance on the duration of behaviours across time bins.

| Behaviour | Comparison | Estimate | SE | z-ratio | p-value |
|---|---|---|---|---|---|
| Flying-Climbing | Before Disturbance – Acute Response | -3.2552 | 0.428 | -7.601 | <0.0001 *** |
| | Before Disturbance – Delayed Response | -2.2179 | 0.415 | -5.342 | <0.0001 *** |
| | Before Disturbance – Recovery | -2.1354 | 0.451 | -4.739 | <0.0001 *** |
| | Acute Response – Delayed Response | 1.0373 | 0.184 | 5.635 | <0.0001 *** |
| | Acute Response – Recovery | 1.1198 | 0.286 | 3.921 | 0.0005 *** |
| | Delayed Response – Recovery | 0.0825 | 0.250 | 0.331 | 0.9876 ns |
| Patrolling | Acute Response – Delayed Response | 1.313 | 0.142 | 9.236 | <0.0001 *** |
| | Acute Response – Recovery | 2.156 | 0.425 | 5.077 | <0.0001 *** |
| | Delayed Response – Recovery | 0.844 | 0.398 | 2.118 | 0.0862 ns |
| Continuous Buzzing | Acute Response – Delayed Response | 2.456 | 0.0897 | 27.373 | <0.0001 *** |
| | Acute Response – Recovery | 0.441 | 0.2519 | 1.751 | 0.1864 ns |
| | Delayed Response – Recovery | -2.015 | 0.2674 | -7.534 | <0.0001 *** |

| | | | | | |
|---|---|---|---|---|---|
| **Leg Raising (Fisher's Test)** | **Acute Response – Delayed Response** | 2.44 | 0.199 | 12.275 | <0.0001 *** |
| **Abdominal Pumping** | **Acute Response – Delayed Response** | 0.665 | 0.0675 | 9.852 | <0.0001 *** |
| | **Acute Response – Recovery** | 2.684 | 0.2879 | 9.325 | <0.0001 *** |
| | **Delayed Response – Recovery** | 2.019 | 0.2726 | 7.407 | <0.0001 *** |
| **Perching** | **Before Disturbance – Acute Response** | -1.89970 | 0.00143 | -1328.670 | <0.0001 *** |
| | **Before Disturbance – Delayed Response** | -0.64494 | 0.00143 | -451.003 | <0.0001 *** |
| | **Before Disturbance – Recovery** | 0.02984 | 0.00143 | 20.866 | <0.0001 *** |
| | **Acute Response – Delayed Response** | 1.25477 | 0.00202 | 620.423 | <0.0001 *** |
| | **Acute Response – Recovery** | 1.92954 | 0.00202 | 954.117 | <0.0001 *** |
| | **Delayed Response – Recovery** | 0.67477 | 0.00202 | 333.709 | <0.0001 *** |
| **Pulse Buzzing** | **Before Disturbance – Acute Response** | -0.465 | 0.230 | -2.023 | 0.1793 ns |
| | **Before Disturbance – Delayed Response** | -0.241 | 0.209 | -1.152 | 0.6573 ns |
| | **Before Disturbance – Recovery** | 0.180 | 0.225 | 0.802 | 0.8536 ns |
| | **Acute Response – Delayed Response** | 0.225 | 0.170 | 1.319 | 0.5507 ns |
| | **Acute Response – Recovery** | 0.646 | 0.191 | 3.381 | 0.0040 ** |

| | | | | | |
|---|---|---|---|---|---|
| | **Delayed Response – Recovery** | 0.421 | 0.165 | 2.551 | 0.0525 * |
| **Grooming** | **Before Disturbance – Acute Response** | 0.0434 | 0.1248 | 0.348 | 0.9856 ns |
| | **Before Disturbance – Delayed Response** | -0.4060 | 0.0979 | -4.147 | 0.0002 *** |
| | **Before Disturbance – Recovery** | 0.2682 | 0.1038 | 2.582 | 0.0483 * |
| | **Acute Response – Delayed Response** | -0.4494 | 0.1097 | -4.096 | 0.0002 *** |
| | **Acute Response – Recovery** | 0.2248 | 0.1192 | 1.886 | 0.2339 ns |
| | **Delayed Response – Recovery** | 0.6742 | 0.0891 | 7.569 | <.0001 *** |

**S6 Table.** Effects of disturbance on the location of behaviours across time bins.

| **Behaviour** | **Bin** | **Mean Duration (s)** | **sd** | **t_stat** | **p_value** |
|---|---|---|---|---|---|
| **Flying-Climbing** | **Before Disturbance** | 0.667 | 1.13 | 1.45 | 0.206 ns |
| | **Acute Response** | 6.89 | 12.7 | 3.30 | 0.00219 ** |
| | **Delayed Response** | 4.46 | 5.68 | 3.60 | 0.00179 ** |
| | **Recovery** | 8.62 | 8.85 | 2.39 | 0.0625 ns |
| **Patrolling** | **Acute Response** | -7.50 | 15.4 | -5.79 | < 0.0001 *** |
| | **Delayed Response** | -0.994 | 8.94 | -0.847 | 0.401 ns |
| | **Recovery** | -1.58 | 5.97 | -0.460 | 0.691 ns |
| **Continuous Buzzing** | **Acute Response** | -7.31 | 7.60 | -11.7 | < 0.0001 *** |
| | **Delayed Response** | -1.48 | 1.02 | -4.38 | 0.0024 ** |
| **Leg Raising** | **Acute Response** | 4.26 | 11.7 | 2.48 | 0.0171 * |
| | **Delayed Response** | -0.33 | 1.20 | -0.48 | 0.678 ns |

| | | | | | |
|---|---|---|---|---|---|
| **Abdominal Pumping** | **Acute Response** | -17.9 | 33.3 | -6.45 | < 0.0001 *** |
| | **Delayed Response** | -18.6 | 17.2 | -12.7 | < 0.0001 *** |
| | **Recovery** | -0.61 | 4.41 | -0.364 | 0.728 ns |
| **Perching** | **Before Disturbance** | -1.84 | 1.20 | -6.68 | < 0.0001 *** |
| | **Acute Response** | -5.27 | 15.40 | -3.29 | 0.00144 *** |
| | **Delayed Response** | -2.30 | 7.31 | -2.44 | 0.0179 * |
| | **Recovery** | -5.04 | 9.28 | -1.44 | 0.201 ns |
| **Pulse Buzzing** | **Before Disturbance** | -1.78 | 11.6 | -0.739 | 0.467 ns |
| | **Acute Response** | -9.28 | 11.9 | -4.94 | < 0.0001 *** |
| | **Delayed Response** | -7.51 | 9.43 | -7.18 | < 0.0001 *** |
| | **Recovery** | -4.96 | 7.55 | -4.64 | < 0.0001 *** |
| **Grooming** | **Before Disturbance** | -4.39 | 8.09 | -4.73 | < 0.0001 *** |
| | **Acute Response** | -1.81 | 5.26 | -2.60 | < 0.0001 *** |
| | **Delayed Response** | -4.56 | 7.28 | -7.23 | < 0.0001 *** |
| | **Recovery** | -2.50 | 6.69 | -3.69 | < 0.0001 *** |

**S7 Table.** Type and count of sound elements in the various data processing steps. **Annotated** – full sample of annotated and positively classified sound events. Used in the rhythmic analysis. **SNR filtered** – Number of sound events passing the 1.5 Signal-to-noise ratio filtering indicating at least a 50% amplitude increase of the analysed sound in comparison to background noise. **Analysed** – a sample size balanced dataset generated by randomly subsampling pulse buzzing sound events. These data were used for the acoustic characterization of the sound types.

| | **Pulse Buzzing** | **Continuous Buzzing** | **Fanning** | **Flying** |
|---|---|---|---|---|
| **Annotated** | 1068 | 123 | 216 | 53 |
| **SNR filtered** | 591 | 76 | 123 | 50 |
| **Analysed** | 250 | 76 | 123 | 50 |

**S8 Table.** Confusion matrix of the random forest output. Columns represent the manually annotated sound types and the rows represent the model assigned sound type.

| Reference / Prediction | Pulse Buzzing | Continuous Buzzing | Fanning | Flying |
|---|---|---|---|---|
| **Pulse Buzzing** | 405 | 15 | 6 | 20 |
| **Continuous Buzzing** | 1 | 36 | 3 | 13 |
| **Fanning** | 5 | 4 | 81 | 9 |
| **Flying** | 0 | 0 | 0 | 2 |

**S9 Table.** A Kolmogorov-Smirnov test results for comparing the distribution of IOI ratios, calculated from the data with the distribution of randomly generated sound times. Only the symmetrical sound pairs were included in the analysis. Sound transition lag was limited to 13 seconds, a 95% cutoff of the overall distribution of IOI intervals. Significant comparisons are in bold.

| Sound pair | n | D | p-value |
|---|---|---|---|
| **Pulse Buzzing** | 1116 | 0.122 | < 0.001 ** |
| **Continuous Buzzing** | 37 | 0.412 | 0.07 ns |
| **flying-climbing _ flying-climbing** | 35 | 0.171 | 0.629 ns |
| **fanning _ fanning** | 229 | 0.102 | 0.2 ns |

**S10 Table.** Pairwise comparison between disturbance types (FO = Foreign Object, MD = Mechanical Disturbance, IB = Intruder Breath) within each time bin (BD = Before Disturbance, AD1 = One min after disturbance, AD2 = Two min after disturbance).

| Behaviour | Comparison | Estimate | SE | t-ratio | p-value |
|---|---|---|---|---|---|
| **Flying-Climbing** | **BD: FO – MD** | 0.0627 | 1.09 | 0.057 | 1.0000 ns |
| | **BD: FO – IB** | −0.6373 | 1.70 | −0.376 | 1.0000 ns |
| | **BD: MD – IB** | −0.7000 | 1.62 | −0.431 | 1.0000 ns |
| | **AD1: FO – MD** | −5.2735 | 1.09 | −4.817 | <.0001 *** |
| | **AD1: FO – IB** | −3.9423 | 1.70 | −2.324 | 0.0634 ns |
| | **AD1: MD – IB** | 1.3312 | 1.62 | 0.820 | 1.0000 ns |
| | **AD2: FO – MD** | −2.8041 | 1.09 | −2.561 | 0.0336 * |
| | **AD2: FO – IB** | −2.4619 | 1.70 | −1.451 | 0.4448 ns |
| | **AD2: MD – IB** | 0.3422 | 1.62 | 0.211 | 1.0000 ns |
| **Patrolling** | **BD: FO – MD** | 1.9086 | 1.11 | 1.721 | 0.2606 ns |
| | **BD: FO – IB** | 0.2120 | 1.72 | 0.123 | 1.0000 ns |
| | **BD: MD – IB** | −1.6966 | 1.64 | −1.031 | 0.9109 ns |
| | **AD1: FO – MD** | -5.1196 | 1.11 | -4.616 | <.0001 *** |
| | **AD1: FO – IB** | -5.0914 | 1.72 | -2.963 | 0.0103 ** |
| | **AD1: MD – IB** | 0.0282 | 1.64 | 0.017 | 1.0000 ns |
| | **AD2: FO – MD** | 0.4835 | 1.11 | 0.436 | 1.0000 ns |
| | **AD2: FO – IB** | 3.2454 | 1.72 | 1.889 | 0.1812 ns |
| | **AD2: MD – IB** | 2.7619 | 1.64 | 1.679 | 0.2843 ns |
| **Continuous Buzzing** | **BD: FO – MD** | -0.642 | 0.964 | -0.666 | 1.0000 ns |
| | **BD: FO – IB** | -1.101 | 1.493 | -0.737 | 1.0000 ns |
| | **BD: MD – IB** | -0.459 | 1.429 | -0.321 | 1.0000 ns |
| | **AD1: FO – MD** | -7.689 | 0.964 | -7.980 | <.0001 *** |
| | **AD1: FO – IB** | -5.706 | 1.493 | -3.823 | 0.0005 *** |
| | **AD1: MD – IB** | 1.983 | 1.429 | 1.387 | 0.5006 ns |
| | **AD2: FO – MD** | -0.847 | 0.964 | -0.879 | 1.0000 ns |
| | **AD2: FO – IB** | -0.978 | 1.493 | -0.655 | 1.0000 ns |
| | **AD2: MD – IB** | -0.131 | 1.429 | -0.091 | 1.0000 ns |
| **Leg Raising** | **BD: FO – MD** | 0.000 | 0.949 | 0.000 | 1.0000 ns |
| | **BD: FO – IB** | 0.000 | 1.470 | 0.000 | 1.0000 ns |
| | **BD: MD – IB** | 0.000 | 1.408 | 0.000 | 1.0000 ns |

| | | | | | |
|---|---|---|---|---|---|
| | **AD1: FO – MD** | -5.871 | 0.949 | -6.187 | <.0001 *** |
| | **AD1: FO – IB** | -6.419 | 1.470 | -4.366 | 0.0001 *** |
| | **AD1: MD – IB** | -0.547 | 1.408 | -0.389 | 1.0000 ns |
| | **AD2: FO – MD** | -5.029 | 0.949 | -5.300 | <.0001 *** |
| | **AD2: FO – IB** | -8.254 | 1.470 | -5.614 | <.0001 *** |
| | **AD2: MD – IB** | -3.225 | 1.408 | -2.291 | 0.0691 ns |
| **Abdominal Pumping** | **BD: FO – MD** | 0.511 | 0.845 | 0.605 | 1.0000 ns |
| | **BD: FO – IB** | -1.859 | 1.309 | -1.420 | 0.4715 ns |
| | **BD: MD – IB** | -2.370 | 1.253 | -1.891 | 0.1802 ns |
| | **AD1: FO – MD** | -0.473 | 0.845 | -0.560 | 1.0000 ns |
| | **AD1: FO – IB** | -0.290 | 1.309 | -0.222 | 1.0000 ns |
| | **AD1: MD – IB** | 0.183 | 1.253 | 0.146 | 1.0000 ns |
| | **AD2: FO – MD** | -1.751 | 0.845 | -2.072 | 0.1188 ns |
| | **AD2: FO – IB** | -1.260 | 1.309 | -0.963 | 1.0000 ns |
| | **AD2: MD – IB** | 0.490 | 1.253 | 0.391 | 1.0000 ns |
| **Pulse Buzzing** | **BD: FO – MD** | -0.349 | 1.21 | -0.288 | 1.0000 ns |
| | **BD: FO – IB** | 0.316 | 1.80 | 0.176 | 1.0000 ns |
| | **BD: MD – IB** | 0.665 | 1.71 | 0.389 | 1.0000 ns |
| | **AD1: FO – MD** | -4.226 | 1.21 | -3.495 | 0.0018 ** |
| | **AD1: FO – IB** | -2.768 | 1.80 | -1.538 | 0.3775 ns |
| | **AD1: MD – IB** | 1.458 | 1.71 | 0.854 | 1.0000 ns |
| | **AD2: FO – MD** | -6.590 | 1.21 | -5.451 | <.0001 *** |
| | **AD2: FO – IB** | -5.658 | 1.80 | -3.144 | 0.0059 ** |
| | **AD2: MD – IB** | 0.932 | 1.71 | 0.546 | 1.0000 ns |
| **Grooming** | **BD: FO – MD** | 0.223 | 1.50 | 0.148 | 1.0000 ns |
| | **BD: FO – IB** | -1.521 | 2.24 | -0.679 | 1.0000 ns |
| | **BD: MD – IB** | -1.743 | 2.12 | -0.821 | 1.0000 ns |
| | **AD1: FO – MD** | 1.150 | 1.50 | 0.765 | 1.0000 ns |
| | **AD1: FO – IB** | -0.272 | 2.24 | -0.122 | 1.0000 ns |
| | **AD1: MD – IB** | -1.422 | 2.12 | -0.670 | 1.0000 ns |
| | **AD2: FO – MD** | -0.336 | 1.50 | -0.224 | 1.0000 ns |

| | | | | | |
|---|---|---|---|---|---|
| | **AD2: FO – IB** | 1.642 | 2.24 | 0.734 | 1.0000 ns |
| | **AD2: MD – IB** | 1.978 | 2.12 | 0.932 | 1.0000 ns |
| **Presence Outside the Brood Area** | **BD: FO – MD** | 2.576 | 0.75 | 3.436 | 0.0022 ** |
| | **BD: FO – IB** | -0.137 | 1.16 | -0.118 | 1.0000 ns |
| | **BD: MD – IB** | -2.712 | 1.11 | -2.440 | 0.0468 * |
| | **AD1: FO – MD** | 0.271 | 0.75 | 0.361 | 1.0000 ns |
| | **AD1: FO – IB** | -0.400 | 1.16 | -0.345 | 1.0000 ns |
| | **AD1: MD – IB** | -0.671 | 1.11 | -0.603 | 1.0000 ns |
| | **AD2: FO – MD** | 0.780 | 0.75 | 1.041 | 0.8980 ns |
| | **AD2: FO – IB** | 4.203 | 1.16 | 3.619 | 0.0011 *** |
| | **AD2: MD – IB** | 3.423 | 1.11 | 3.078 | 0.0071 ** |
