# Supplementary material for: Colony defence in bumblebees (Bombus terrestris)
Source: PLoS One. 2025 Dec 3;20(12):e0335136. doi: 10.1371/journal.pone.0335136 (PMC12674513; doi:10.1371/journal.pone.0335136)
Supplement: Attachment — Submitted filename: Rebuttal letter.docx [file pone.0335136.s003.docx]

Please see below the comments from the reviewers in green, and our responses in black.

Reviewer #1: Thank you for giving me the opportunity to review this manuscript, “Colony defence in bumblebees (Bombus terrestris).”

In this paper, the authors characterise the behavioural response of bumblebee workers to the disturbance of their nest, analysing bout video and audio data. The ethogram created by the authors was detailed, and the methods of disturbance were well-selected: they should be commended for these, as well as their thorough analysis of the behaviours that they recorded. The discussion of these behaviours and their implications is also strong. Overall, I think the paper is good, and would recommend it for publication should my comments (and those of other reviewers) be addressed. I would like to highlight in particular that there are issues with the figures, at least in the document that I downloaded, in terms of resolution, quality and consistency: this does need to be corrected.

We thank Reviewer #1 for this positive assessment of our work and their careful reading. Below are detailed answers to their comments.

MAIN COMMENTS

1. This manuscript would benefit from a careful read-through for grammar and syntax issues: commas in particular are frequently in the wrong place in sentences and that means things don’t quite read smoothly.

We have read through the paper and corrected any mistakes we found.

2. I would consider pooling all information about the bees for all parts of the experiment into one section of the methods, e.g. we hear first about the four larger colonies (and also the single worker set ups) and then much later on about the microcolonies. There also isn’t any information as to how the microcolonies were set up and established, as there was for the larger colonies, so it would be better to keep this information all together. Then, just reiterate when you get to each different experiment that you used the larger colonies/microcolonies/single worker set-ups.

Thanks for your suggestion. We have pooled the information as suggested, and added some reminders of the type of set-ups used in relevant places.

3. I don’t know whether it’s the system compressing the figure images oddly, but they seem very low resolution and grainy in the file that I downloaded (apart from Fig. 5 which for some reason alone is nice and crisp). In some places the text is actually impossible to read (especially in Fig. 3 and 4). From what I can tell they are probably good figures, and that’s nice to see, but the resolution issues absolutely need to be fixed. There also doesn’t seem to be much consistency in terms of formatting or fonts across figures, which is confusing.
The low resolution is only a quirk of the online pdf builder. We also checked and corrected the figures for consistency.

MINOR COMMENTS

Line 20: “specific associations of behaviours: - what does this mean? I’m unclear. Do you mean whether individual bees are associated with a particular behaviour, or that they produce idiosyncratic behaviours?

We meant whether some behaviours are produced together and by the same individual, or in other words if bees responding with behaviour A also do behaviour B. We’ve replaced “associations” with “combinations”, we hope that makes it clearer.

Line 21: Again, “associated” is not right here. “Performed” might be better, or “produced”?

We replaced “associated” with “performed” as suggested.

Line 25: Would “potentially signalling the threat to other colony members” fall under “preparing members for a response”? As written, it feels vague, so I would perhaps remove this.

By “preparing members”, we meant the abdominal pumping that could be a way to increase metabolic activity, whereas “signalling” referred to buzzing. We agree that this sentence is a bit vague, this is due to the length constraint of the abstract and the fact that we have to remain speculative, since we did not prove the function of each behaviour. We think, however, that it is important to mention these as being the most likely interpretations of our results.

Line 32: This is probably not how I would go about describing an animal to open a scientific paper, as it’s a little casual. I would rewrite the whole sentence to something like this: “The relatively docile temperament of bumblebees, coupled with their colourful fur, belies their capacity to defend their nests fiercely from danger. As their venom…”
This opening sentence is catchy on purpose, we like it that way :).

Line 39: Or cuckoo bumblebees?

Cuckoo bees were implicitly included in the “parasitic queens”, but we now mention them more directly.

Line 41: I would avoid using nectar robbing here as this term is most frequently used for a specific behaviour where bumblebees bite through the corolla of a flower to steal nectar. I would term this as “nectar theft” or “resource theft” to avoid confusion.

Done.

Line 46: This might read better as: “Bombus terrestris guard
bees are surprisingly permissive to sterile non-nestmates, most often merely antennating them for longer. They also display self-grooming upon encountering them.”
(Do you mean that the guard bees groom themselves when they encounter the non nestmate?)

We have modified this sentence as suggested. And yes, this is their reaction according to the paper by Blacher et al.

Line 54: Should this reference be written in the same style as the others?
We’ve modified this sentence to cite the reference at the end only.

Line 72: ”and very obvious to anyone who ever bothered them” - again, this reads a little too casually, I would remove or modify by swapping “bothered” to “disturbed”.

“disturbed” being already used 2 words later, we decided to keep “bothered”.

Line 82: Again, “produce specific associations of behaviours”sounds wrong: “…if individual bees are associated with specific behaviours” would be better. Unless you mean to talk about behavioural syndromes, here?

We modified this sentence to: “if some behaviours are produced in combinations by single individuals, as a correlated pattern or even a sequence”.

Line 92: Between which months/years?

This information has been added.

Line 149: How much time?

We couldn’t track these bees because they went out of view, which means that we also couldn’t track how long they went missing.

Line 153: “For collecting the audio imprint of bees” is somewhat unusual wording, “To collect audio from the bees” would be better.
Changed as suggested.

Line 156: The methods section is quite long, so I think you should just once again clarify here that these were the single-worker set-ups that you established (at first, I wondered how you managed to seperate out the noise from one bee from the whole group, and had to go back to realise these were individual worker set-ups!)

Done.

Line 392: if the distinction is that these behaviours occurred mostly during the acute phase, I would maybe just call this “Acute behavioural responses”.
Line 406: Again, I would perhaps terms these “persistent behavioural responses” or something - saying they are related to a “sustained alerted state” might have some assumptions in it? It also contrasts better to “acute behavioural responses”.

We re-named these categories as suggested.

Line 403: I don’t quite follow the logic here. Why would continous buzzing being performed in the brood area and leg raising being performed outside of it mean that these are transient reactions? This sentence should be put before the descriptions for the locations for these behaviours.

We moved this sentence accordingly.

Line 419: “Delayed responses” might be clearer.

Changed.

Line 598: “digged-out” -> “dug out” (but good discussion of this limitation).

Corrected.

Reviewer #2: I really enjoyed reading this manuscript. It is a well-designed and elegant study that provides the first quantitative analysis of holistic defensive behaviour in this important model system. Everything was clearly described, and I couldn't identify anywhere where the manuscript was confusing or unclear. The results will be of broad interest to bumblebee biologists, as well as those interested in defensive behaviour in social insects, I congratulate the authors on a really nice piece of work

Thank you!
